# Asthma and atopic dermatitis as risk factors for rheumatoid arthritis: a bidirectional mendelian randomization study

**DOI:** 10.1186/s12920-023-01461-7

**Published:** 2023-03-03

**Authors:** Chuiji Chen, Le Su, Wenhao Duan, Yansen Zheng, Dianzhong Zhang, Yucai Wang

**Affiliations:** 1 Department of Orthopedic Surgery, Tangdu Hospital, Fourth Military Medical University, No. 1 of Xinsi Road, Baqiao District, 710038 Xi’an City, Shaanxi Province China; 2 Department of Orthopedic Surgery, The Fourth People’s Hospital of Zibo, No. 210 of Shanquan Road, Zhangdian District, 255051 Shandong, Shandong Province China; 3 Xi’an Medical University, No. 1 of Xinwang Road, Weiyang District, 710021 Xi’an City, Shaanxi Province China; 4 School of Medicine, Yellow River Institute of Science and Technology, 666 Zijing Shan South Road, 450015 Zhengzhou City, Henan Province China

**Keywords:** Asthma, Atopic dermatitis, Rheumatoid arthritis, Genetics, Mendelian randomization

## Abstract

**Background:**

Previous observational studies have shown an association between asthma, atopic dermatitis (AD) and rheumatoid arthritis (RA). However, the bidirectional cause-effect chain between asthma and AD and RA has not been proven yet.

**Methods:**

We performed bidirectional two-sample Mendelian randomization (TSMR) and selected single nucleotide polymorphisms (SNPs) associated with asthma, AD, and RA as instrumental variables. All of the SNPs were obtained from the latest genome-wide association study in Europeans. Inverse variance weighted (IVW) was the main method used in MR analysis. MR-Egger, weighted model, simple model, and weighted median were used for quality control. The robustness of the results was tested by sensitivity analysis.

**Results:**

Asthma was found to be the largest effect size for RA susceptibility using the IVW method (OR, 1.35;95%CI, 1.13–1.60; *P*, 0.001), followed by AD (OR, 1.10;95%CI, 1.02–1.19; *P*, 0.019). In contrast, there was no causal relationship between RA and asthma (IVW: *P* = 0.673) or AD (IVW: *P* = 0.342). No pleiotropy or heterogeneity was found in the sensitivity analysis.

**Conclusion:**

Findings from this study showed a causal relationship between genetic susceptibility to asthma or AD and increased risk of RA, but do not support a causal relationship between genetic susceptibility to RA and asthma or AD.

**Supplementary Information:**

The online version contains supplementary material available at 10.1186/s12920-023-01461-7.

## Introduction

Rheumatoid arthritis (RA) is a systemic autoimmune disease characterized by joint inflammation and destruction, followed by systemic inflammation. An estimated 0.4–1.3% of people in the world are affected by this disease, and its incidence rate in women is 2 to 4 times higher [1]. The occurrence of RA will not only affects the quality of life of patients but also brings great economic losses to society [2, 3]. RA is a disease caused by multiple factors, involving a complex interaction between genetic predisposition and environmental triggers. Smoking has been implicated as a major risk factor for RA as a result of the production of anti-citrullinated peptide antibodies (ACPA) [4, 5]. Moreover, the lifetime risk of RA is significantly higher for individuals who carry certain human leukocyte antigen (HLA) alleles [6].

To prevent the occurrence and development of RA, other risk factors are receiving increasing attention from scholars, such as allergic diseases. An observational study has shown that patients with allergic diseases, particularly asthma and allergic rhinitis, are at significantly increased risk for RA [7]. However, a cross-sectional study noted a higher prevalence of RA in non-asthmatic patients compared to asthmatic patients [8]. Moreover, Lu et al. [9] noted in a study that atopic dermatitis (AD) increases the risk of developing RA. While in contrast, Hilliquin et al. [10] suggested that atopic reactivity may have a preventive effect on the development of RA. Meanwhile, several reports have suggested that patients with RA have less atopic disease than patients in normal controls [11–13]. In contrast, Kero et al. [14] reported a significantly higher cumulative incidence of asthma in children with RA than in non-RA children. A study showed an association between RA and an increased risk of allergic diseases such as asthma and allergic rhinitis [15]. In light of the controversial association between the results of these studies, and the limitations of observational studies, further research is needed to determine this relationship. At the same time, studies examining the link between allergic diseases and RA at the genetic level have yet to emerge.

Mendelian randomization (MR), an epidemiological method, has been widely executed to evaluate the potential causal associations between exposures and outcomes [16, 17]. In MR analyses, using genetic variants as instrumental variables (IVs) could minimize confounders’ inverse causations or effects [18, 19]. Of note, a large number of recent Genome Wide Association Study (GWAS) data on allergic disease [20] and RA allow the use of MR analysis to explore associated disease causality [21–23]. Therefore, to fill the limitations of the current study, we performed a bidirectional TSMR analysis aimed at examining whether there is a causal association between allergic diseases (asthma and AD) and RA, as well as determining the direction of causality.

## Data and Methods

### Study design overview

**Fig. 1:**
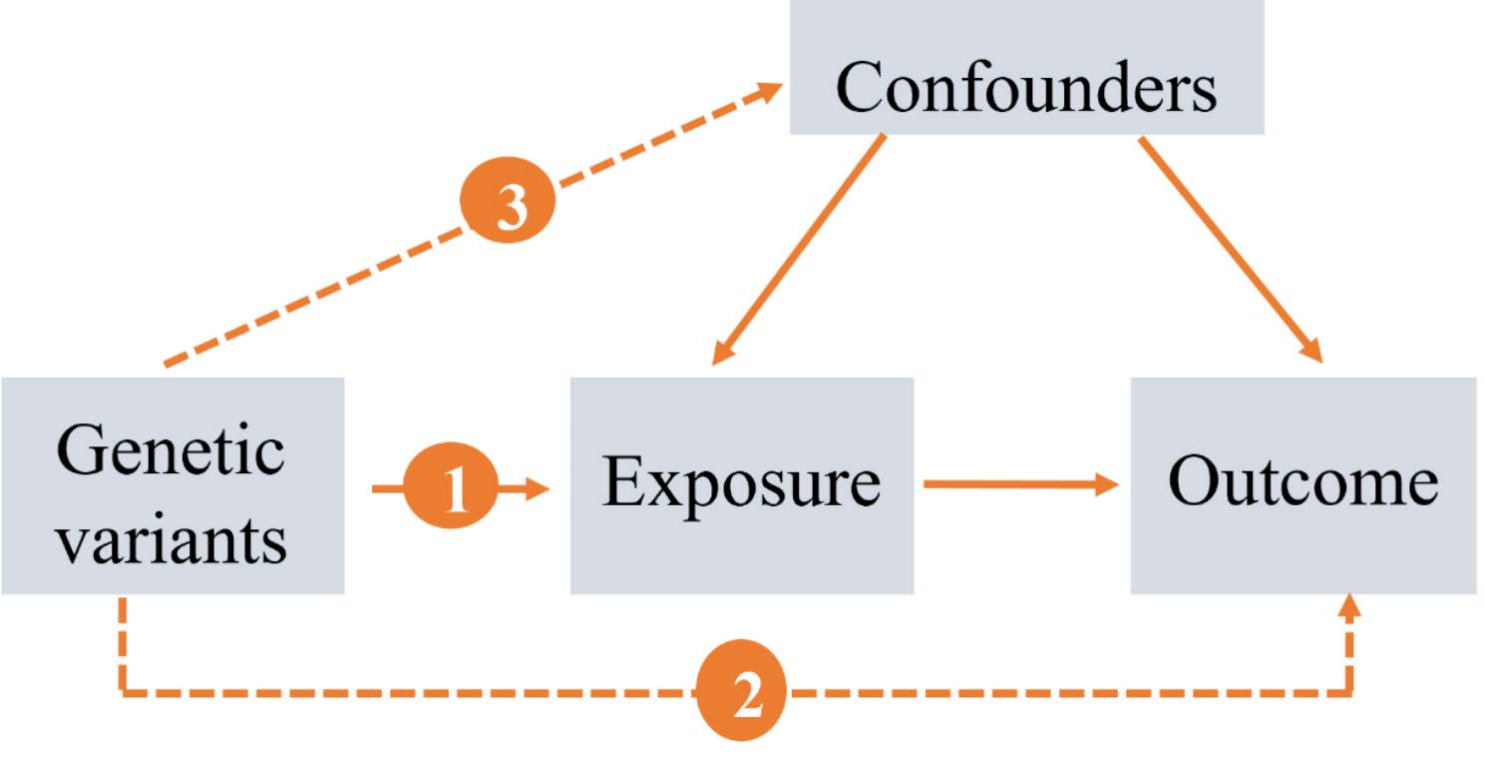
The rationale of Mendelian randomization 1 represents the instrumental variables (IVs) that are strongly associated with the exposure; 2 indicates the IVs must influence the outcome only through the exposure; 3 shows the IVs must not associate with con-founders

We executed a bidirectional TSMR analysis to assess the bidirectional causal effects of asthma or AD and RA. We applied previously identified genetic variants (single-nucleotide polymorphisms, SNPs) from published data or the Genome Reference Consortium to estimate their potential causal effects of exposures on outcomes. The valid MR analyses were structured to define the following three key assumptions: [1] genetic variants are strongly associated with the studied exposures, [2] exposures and outcomes are independent of any known confounders, and [3] genetic variants do not affect the outcomes through alternative pathways except the relevant assumption. The relationships between the exposures and outcomes are illustrated in Fig. 1.

### Date sources

The summary statistics of the GWAS for asthma, AD, and RA were currently obtained from the IEU OpenGWAS (https://gwas.mrcieu.ac.uk/). There are 56,167 cases and 352,255 controls for asthma; 7,024 cases and 198,740 controls for AD; and 2,843 cases and 5,540 controls for RA. All cases were confirmed by clinical laboratory testing, physician diagnosis, or self-reported. To reduce outcome bias from race-related confounders, the study was limited to the European population. **Table S1** shows detailed information on our data used.

### Instrumental variables

At the beginning of our study design, we selected appropriate SNPs as IVs, which must be robustly associated with exposures (*P* < 5 × 10^− 8^). SNPs should be restricted by low linkage disequilibrium (LD, r^2^ < 0.01, 5,000 kb) using clumping. In addition, we excluded palindromic SNPs whose minor allele frequency (MAF) of outcomes was less than 0.01.

### Mendelian randomization analyses

All analyses were executed in R version 4.2.0 using the TwoSampleMR package 0.5.6. After selecting appropriate SNPs of exposures, the inverse-variance weighted (IVW) analysis was chosen as the primary approach to evaluate the causal-and-effect relationship. Then, we added a series of MR analyses including Mendelian randomization-egger (MR-Egger), Weighted median (WM), Weighted mode, and Simple mode methods. When performing MR approaches, *P* < 0.05 was regarded as suggestive evidence for potential association. The odd ratio (OR) and standard error (SE) were calculated to show effect sizes. In addition, the IVW method and MR-Egger regression were used to investigate the presence of heterogeneity in the results, which was quantified using Cochran’s Q-test [24]. MR-Egger regression is also used to determine the likelihood of pleiotropy, with the intercept term indicating potential horizontal pleiotropy [25]. Meanwhile, we also used the “leave-one-out” method to remove single SNP which has a significant independent influence on the MR method [26].

## Results

### Strength of genetic instruments

Based on the above criteria for MR analyses, 53 SNPs associated with asthma, 25 SNPs associated with AD, and 40 SNPs correlated with RA were used as instrumental variables for subsequent analysis. All of these IVs had F-values greater than 10, indicating that the bias of these instrumental variables did not directly affect the assessment of causal effects (**Table S2**).

### Effect of asthma and AD on RA

The effects of each SNPs in asthma and AD on RA can be found in Table 1. We found that the causal relationship between asthma or AD and RA differed among the five MR methods. MR results for the IVW, MR-Egger, and WM methods showed a significant connection between asthma and RA (IVW, OR = 1.35, 95% CI = 1.13–1.60, *P* = 0.001; MR-Egger, OR = 2.62, 95% CI = 1.20–5.72, *P* = 0.020; WM, OR = 1.29, 95% CI = 1.01–1.66, *P* = 0.042) (Table 1). For AD, the results of IVW method can support this causal relationship (OR = 1.10, 95% CI = 1.02–1.19, *P* = 0.019) **(**Table 1**)**. The MR-Egger’s analysis showed no underlying horizontal pleiotropy (asthma: *P* = 0.094, AD: *P* = 0.450) (Table 2). Cochran’s Q test (Table 2) showed no heterogeneity in the risk of asthma or AD and RA. Furthermore, we conducted a “leave-one-out analysis” and found that none of the SNPs strongly influenced the overall effect of asthma or AD and RA (**Fig. S1**).

**Table 1:** Effects of genetically predicted Asthma and AD on the risk of RA in the MR analysis

Exposure	Outcome	No. SNPs	MR methods	*P*	OR	95%CI
Asthma	RA	51	MR Egger	0.020	2.62	1.20–5.72
			WM	0.042	1.29	1.01–1.66
			IVW	0.001	1.35	1.13–1.60
			Simple mode	0.477	1.23	0.70–2.16
			Weighted mode	0.451	1.24	0.71–2.15
AD	RA	24	MR Egger	0.706	1.03	0.87–1.23
			WM	0.056	1.12	1.00-1.25
			IVW	0.019	1.10	1.02–1.19
			Simple mode	0.230	1.13	0.93–1.36
			Weighted mode	0.171	1.12	0.96–1.31

MR, Mendelian randomization analysis; SNP, Single nucleotide polymorphism; IVW, Inverse variance weighted; WM, Weighted median; AD, Atopic dermatitis; RA, Rheumatoid arthritis

**Table 2:** Sensitivity analysis of asthma and AD with RA by different analysis methods

Exposure	Outcome	MR methods	Cochran Q statistic	Heterogeneity *P*	Pleiotropy *P*
Asthma	RA	MR Egger	56.242	0.222	0.094
		IVW	59.584	0.166	
AD	RA	MR Egger	21.312	0.502	0.450
		IVW	21.903	0.526	

MR, Mendelian randomization analysis; IVW, Inverse variance weighted; AD, Atopic dermatitis; RA, Rheumatoid arthritis

### Effect of RA on asthma and AD

As shown in Tables 3 and 40 instrumental variables were included in the reverse MR analysis. To determine the correlation between RA and asthma or AD, we performed a reverse MR analysis and the IVW method showed no correlation between RA and asthma (IVW: *P* = 0.673). Likewise, there is no causal relationship between RA and AD (IVW: *P* = 0.342). Sensitivity analyses and tests of heterogeneity did not indicate potential horizontal pleiotropy and significant heterogeneity (Table 4). No single SNP strongly affected the overall outcome of RA on asthma and AD as demonstrated by the “leave-one-out” sensitivity analysis (**Fig. S2**).

**Table 3:** Effects of genetically predicted RA on the risk of Asthma and AD in the MR analysis

Exposure	Outcome	No. SNPs	MR methods	*P*	OR	95%CI
RA	Asthma	40	MR Egger	0.694	1.02	0.94–1.09
			WM	0.396	1.01	0.98–1.05
			IVW	0.673	1.01	0.98–1.03
			Simple mode	0.258	1.04	0.97–1.12
			Weighted mode	0.252	1.04	0.97–1.11
RA	AD	40	MR Egger	0.750	1.03	0.88–1.19
			WM	0.249	1.05	0.97–1.14
			IVW	0.342	1.03	0.97–1.09
			Simple mode	0.533	1.06	0.88–1.29
			Weighted mode	0.304	1.11	0.91–1.35

MR, Mendelian randomization analysis; SNP, Single nucleotide polymorphism; IVW, Inverse variance weighted; WM, Weighted median; AD, Atopic dermatitis; RA, Rheumatoid arthritis

## Discussion

In the present study, we assessed the bidirectional causal relationship between asthma, AD, and RA. By using a two-sample MR approach and using GWAS summary statistics, the results showed a positive causal relationship between asthma, AD and RA. In reverse MR analysis, no causal relationship was observed between RA and asthma or atopic dermatitis. These findings suggested that the three diseases may have similar pathogenesis.

**Table 4:** Sensitivity analysis of RA with asthma and AD by different analysis methods

Exposure	Outcome	MR methods	Cochran Q statistic	Heterogeneity *P*	Pleiotropy *P*
RA	Asthma	MR Egger	23.869	0.964	0.786
		IVW	23.944	0.972	
RA	AD	MR Egger	58.907	0.370	0.969
		IVW	58.908	0.406	

MR, Mendelian randomization analysis; IVW, Inverse variance weighted; AD, Atopic dermatitis; RA, Rheumatoid arthritis

Previous epidemiological studies have examined the associations between asthma, AD, and RA. Several case-control studies have identified asthma as a possible risk factor for RA, but with the potential for recall bias [27–29]. In addition, retrospective cohorts have analyzed the overall risk of asthma and RA [7, 8, 30–32]. Studies using administrative datasets have also reported an association between asthma and RA risk, but lack data on smoking or serum status with RA [30–32]. Certainly, in a large national study in Taiwan based on billed claims data, RA was associated with a 2-fold increased risk of asthma compared to controls [11]. Furthermore, studies on AD have published different views, and several previous epidemiological studies have shown no association between AD and RA [10, 33, 34]. However, recent studies point to a correlation between AD and RA and that AD may promote the development of RA [15, 29, 35]. Our study was based on the largest available GWAS dataset and restricted the population to individuals of European ancestry to avoid bias on account of small sample size or ethnic differences. The results showed that both asthma and AD are related to an increased risk of RA and that there is no reverse causality between them, suggesting that the previously observed controversial results may be attribute to confounding factors or ethnic differences.

The underlying mechanisms of these associations are poorly understood, but there are still some mechanisms that can be used to explain their possible connection. In the first place, some common immune pathogenesis between not only asthma but also AD and RA. T helper 17 (TH17) cells are one of the pro-inflammatory T helper cell subsets and their increased activity plays a big role in the development of RA [36]. Interleukin 17 (IL-17), an inflammatory factor produced by TH17 cells, has increased expression in AD patients compared to healthy subjects [37]. Of course, studies also suggested that increased TH17 activity and increased IL-17 expression play an important role in the development of airway inflammation in asthma by inducing Th2-interrelated eosinophilia and airway Mucin 5AC expression, as well as increased airway hyperresponsiveness [38–42]. This immune pathway is also thought to be involved in the pathogenesis of RA, as IL-17 expression and TH17 activity are increased in RA patients compared to non-RA individuals [43, 44]. In the second place, studies have pointed out that asthma and RA may have overlapping genetic predispositions, and certain genetic variants in immune-related genes are associated with increased susceptibility to asthma and RA, such as HLA-DRB1 [45, 46], CD40L, and CD86 [47]. Interestingly, certain genes (such as HLA-DRB1 and PTPN22) have been confirmed to be associated with AD and RA [48–50], but no common susceptibility locus have been identified for these two diseases. Therefore, further studies are needed to explore this potential explanation.

In addition, several environmental factors can be used to explain this association of asthma or AD and RA, for example, smoking can contribute to increased inflammation in the lower respiratory tract through a variety of mechanisms and is a predisposing factor for asthma [51]. Smoking also induces the release of peptidylarginine deiminase 2 and enzyme 4 from lung phagocytes, which can convert endogenous proteins into guanylated autoantigens [5]. These guanylated autoantigens, in turn, stimulate the development of anti-citrullinated peptide antibodies in genetically susceptible individuals and may eventually trigger a chronic inflammatory response in synovial junctions, leading to RA development [6, 52]. Toxic substances produced by smoking (e.g., nicotine and carbon monoxide) disrupt skin barrier function, and these substances disrupt blood flow and oxygenation of the skin [53]. These skin disorders and associated subcutaneous structures allow the penetration of allergens into the skin, leading to AD [54]. Thus, smoking is a causative factor in their communication. These possible assumptions can provide theoretical support for our research results.

The main strength of this study is MR analysis; while randomized controlled trials (RCTs) can provide the most convincing evidence, they involve many ethical issues and have high financial costs. For observational studies, which are adjusted for other relative variables, the undetected bias cannot be ignored. Hence, MR provides the most convincing results. Bias due to confounding and reverse sources can be reduced by MR. To minimize potential violations of MR assumptions, we also performed continuous sensitivity analyses and detected any outliers by radial MR analysis.

Nevertheless, the limitations of our MR analyses also need to be acknowledged. Firstly, our results of MR analyses were mainly focused on the European population to reduce the ethnic confounding. Hence, reliable datasets on non-European or mixed population are urgently needed, because we need to break the link correlated with particular genes of the local environment. Secondly, potential pleiotropy could not be fully ruled out, resulting in three hypotheses that could not be accurately evaluated. However, sensitivity analyses were performed using multiple methods to obtain consistent results, which makes the results of this study reassuring.

## Conclusion

Overall, we used two-sample MR analyses to describe the bidirectional causality between asthma, AD, and RA, focusing on filling a gap in knowledge of this causal chain. The results of MR Analysis provide strong evidence for a positive causal relationship between asthma and AD and RA. Nevertheless, more large-scale studies are needed to support our findings and explore the specific mechanisms involved.

## Electronic supplementary material

Below is the link to the electronic supplementary material.

Supplementary Material 1

Supplementary Material 2

Supplementary Material 3

Supplementary Material 4

## Data Availability

As data for this study was acquired via a database (244,991,502,576 genetic associations from 42,335 GWAS summary datasets), the authors applied a broad consent to allow research participants to query and download a broad range of their data (https://gwas.mrcieu.ac.uk/).
