# Supplementary material for: Asthma and atopic dermatitis as risk factors for rheumatoid arthritis: a bidirectional mendelian randomization study
Source: BMC Med Genomics. 2023 Mar 3;16:41. doi: 10.1186/s12920-023-01461-7 (PMC9985208; doi:10.1186/s12920-023-01461-7)
Supplement: Supplementary file 1 — Supplementary Material 1 [file 12920_2023_1461_MOESM1_ESM.docx]

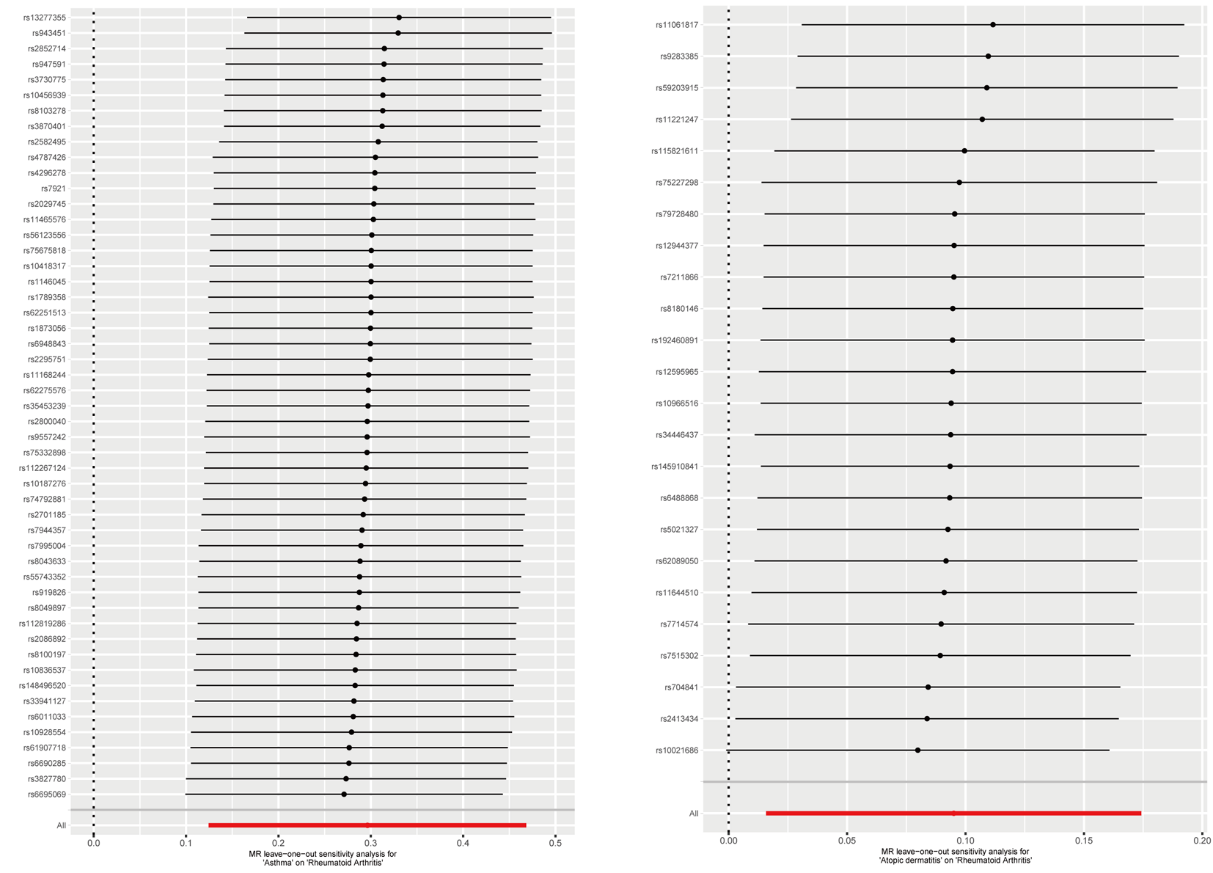
(A) (B)

Fig. S1. A, “Leave-one-out” sensitivity analysis of causal effects of asthma on rheumatoid arthritis. B, “Leave-one-out” sensitivity analysis of causal effects of atopic dermatitis on rheumatoid arthritis.
