# Supplementary material for: Asthma and atopic dermatitis as risk factors for rheumatoid arthritis: a bidirectional mendelian randomization study
Source: BMC Med Genomics. 2023 Mar 3;16:41. doi: 10.1186/s12920-023-01461-7 (PMC9985208; doi:10.1186/s12920-023-01461-7)
Supplement: Supplementary file 2 — Supplementary Material 2 [file 12920_2023_1461_MOESM2_ESM.docx]

(A)
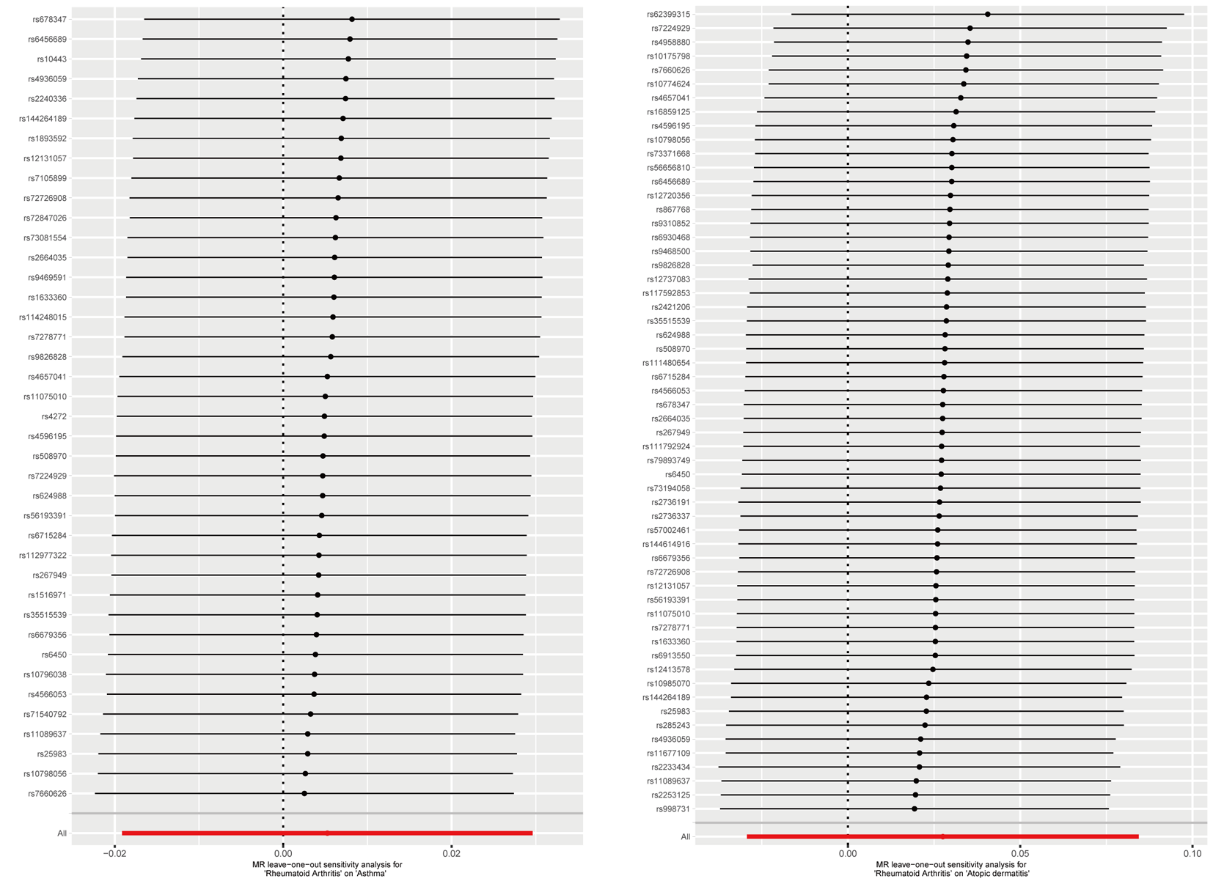
 (B)

Fig. S2. A, “Leave-one-out” sensitivity analysis of causal effects of rheumatoid arthritis on asthma. B, “Leave-one-out” sensitivity analysis of causal effects of rheumatoid arthritis on atopic dermatitis.
